# Supplementary material for: Structural mechanism of proton conduction in otopetrin proton channel
Source: Nat Commun. 2024 Aug 23;15:7250. doi: 10.1038/s41467-024-51803-x (PMC11343839; doi:10.1038/s41467-024-51803-x)
Supplement: Supplementary file 1 — Supplementary Information [file 41467_2024_51803_MOESM1_ESM.pdf]

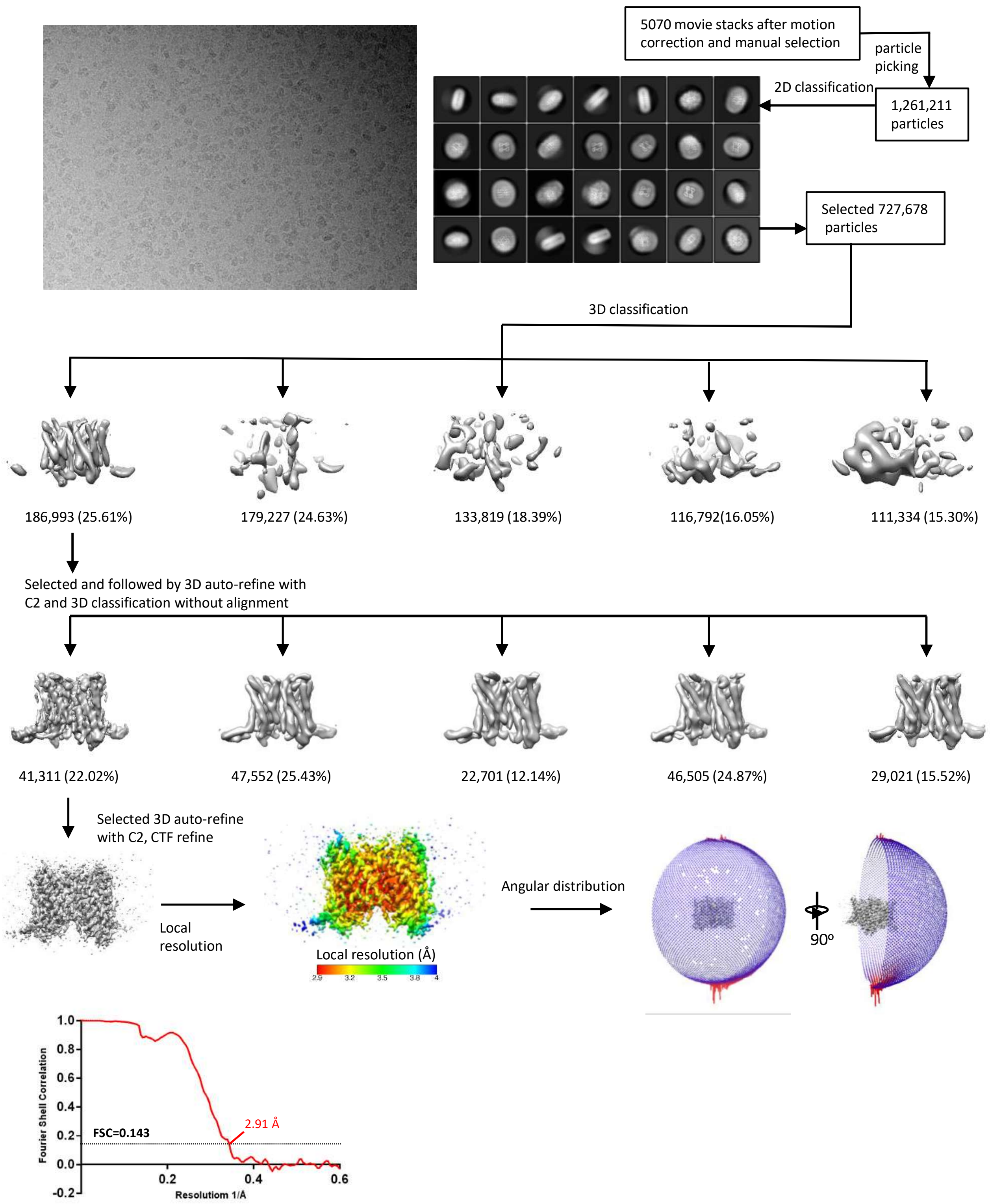

Supplementary Fig. 1. Cryo-EM data processing scheme of CeOTOP8 at pH 5.0.

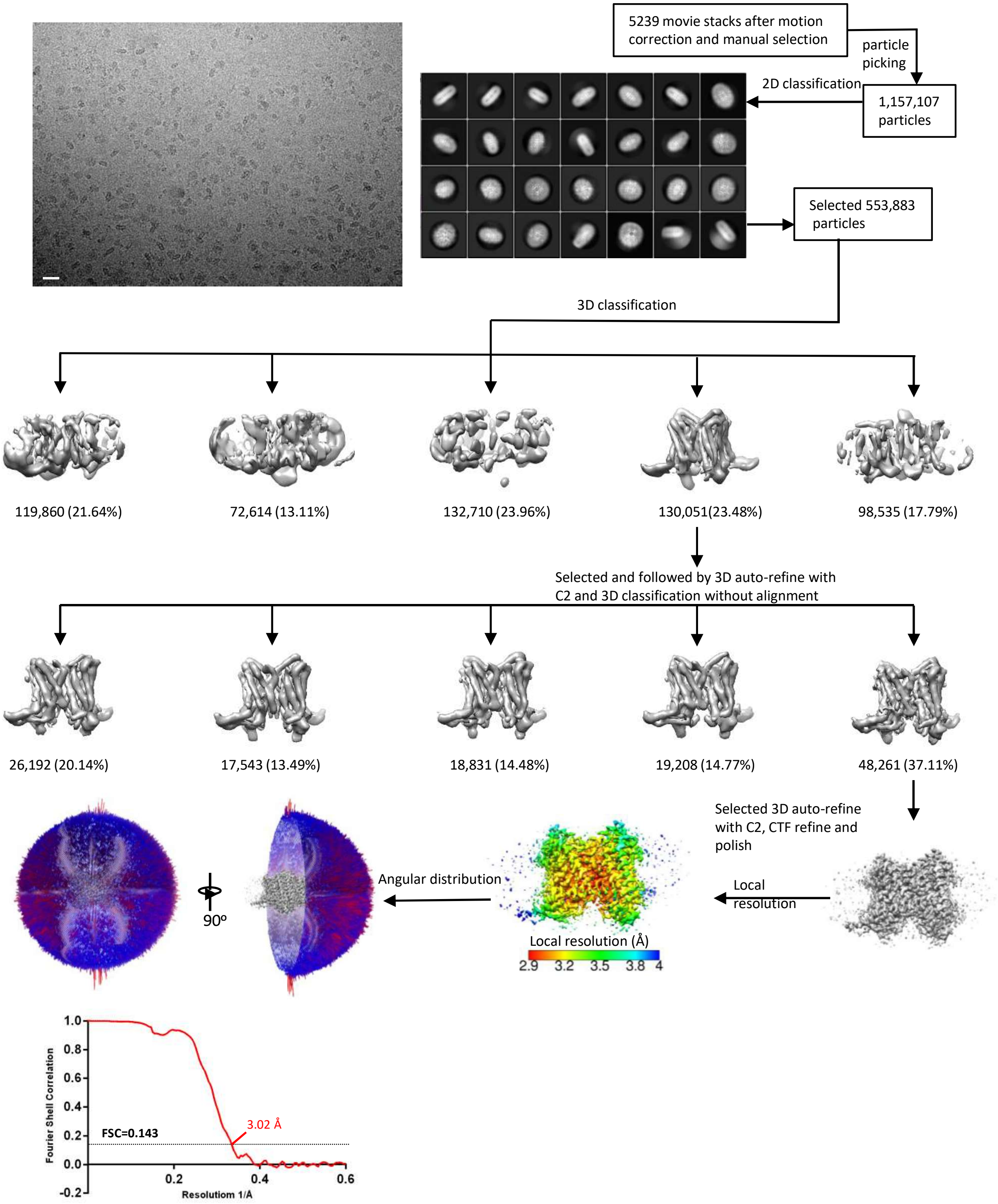

Supplementary Fig. 2. Cryo-EM data processing scheme of CeOTOP8 at pH 8.0.

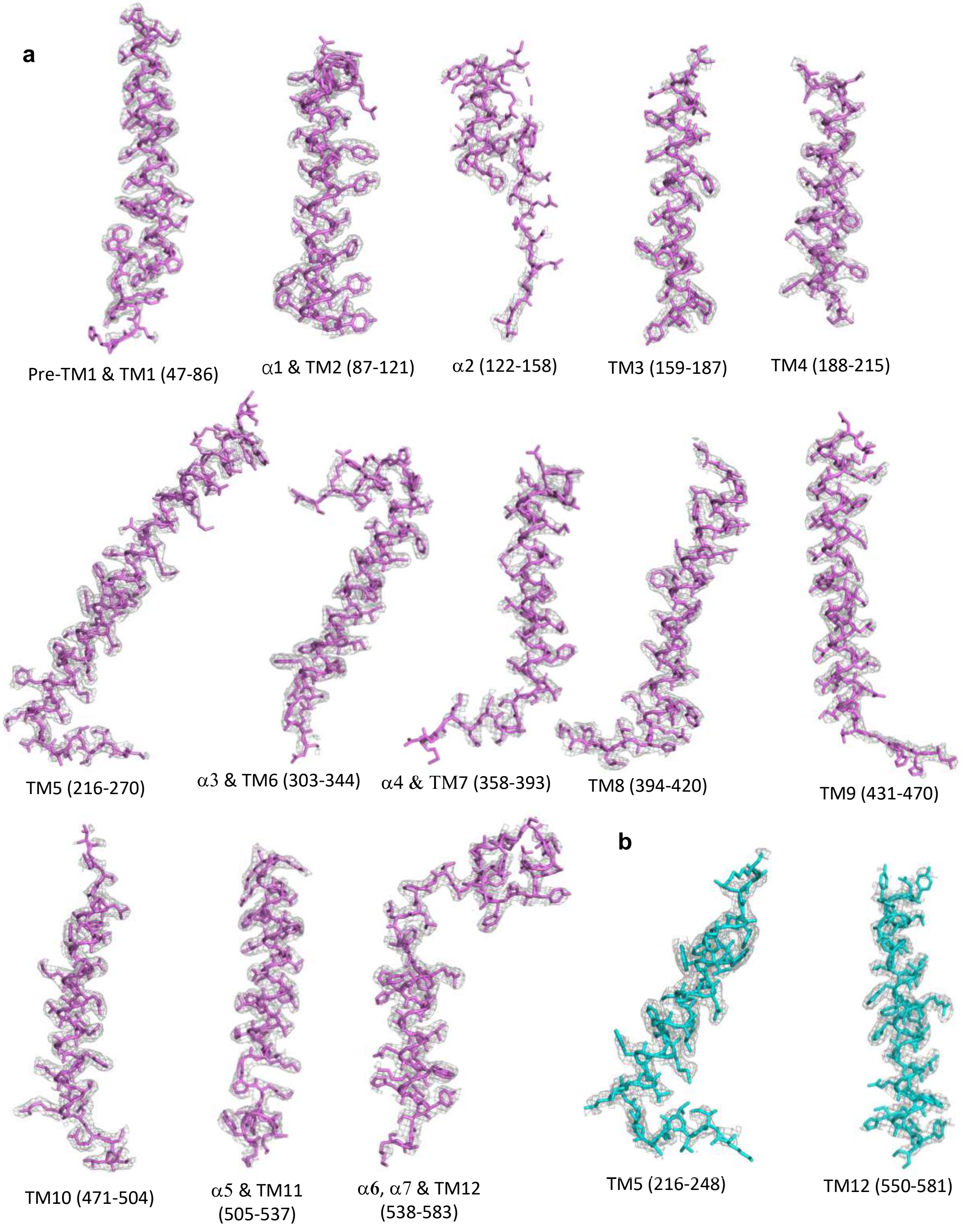

Supplementary Fig. 3. Sample density maps of CeOTOP8.
(**a**). Density maps of CeOTOP8 structure obtained at pH 8.0. All density is contoured at 5σ.
(**b**). Density maps of TMs 5 and 12 from CeOTOP8 structure obtained at pH 5.0. Density is contoured at 5σ.

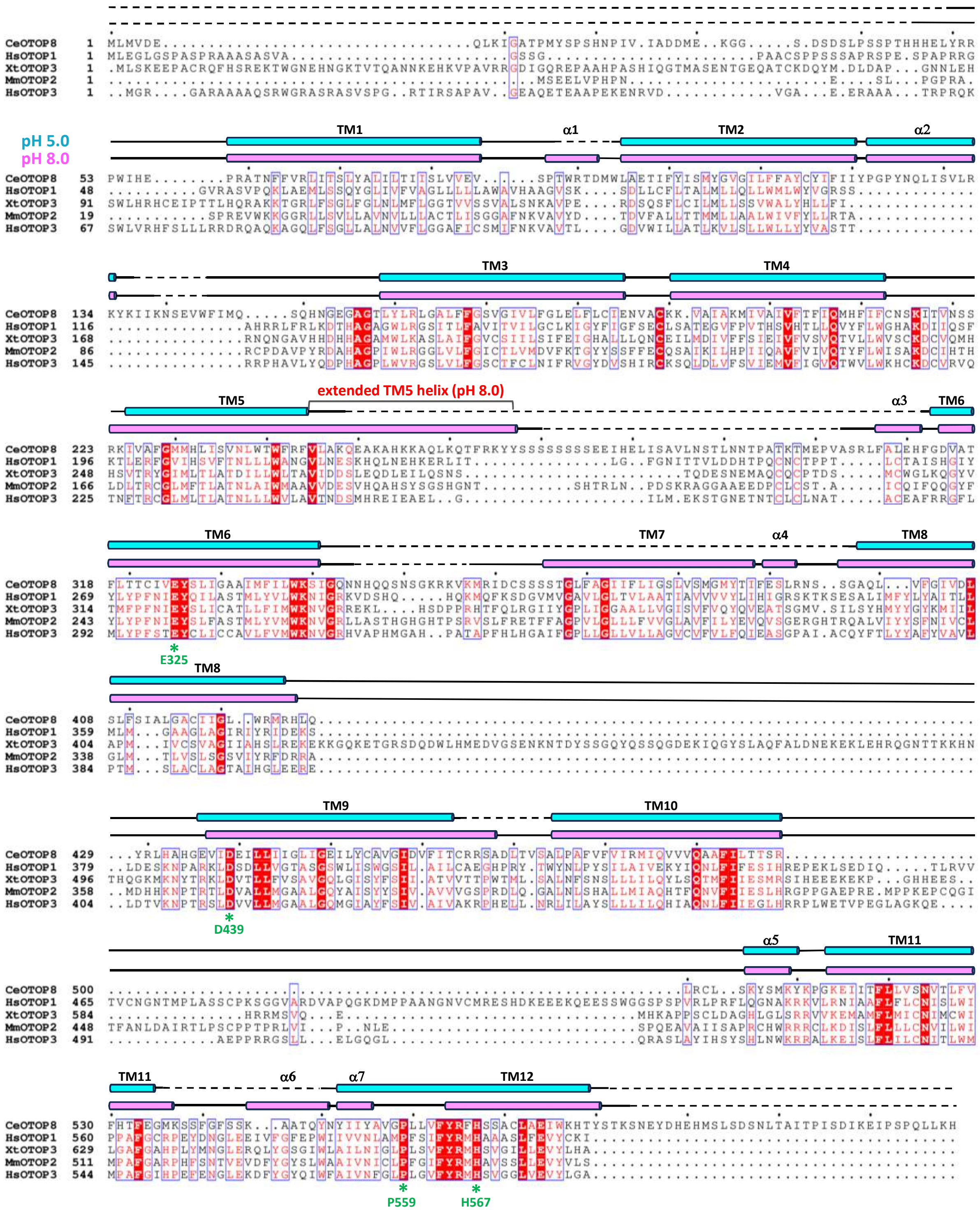

Supplementary Fig. 4. Sequence alignment and secondary structure assignments of CeOTOP8. Conserved residues important for proton conduction are marked by green asterisks. The secondary structure assignments are colored cyan and violet for structures determined at pH 5.0 and 8.0, respectively.

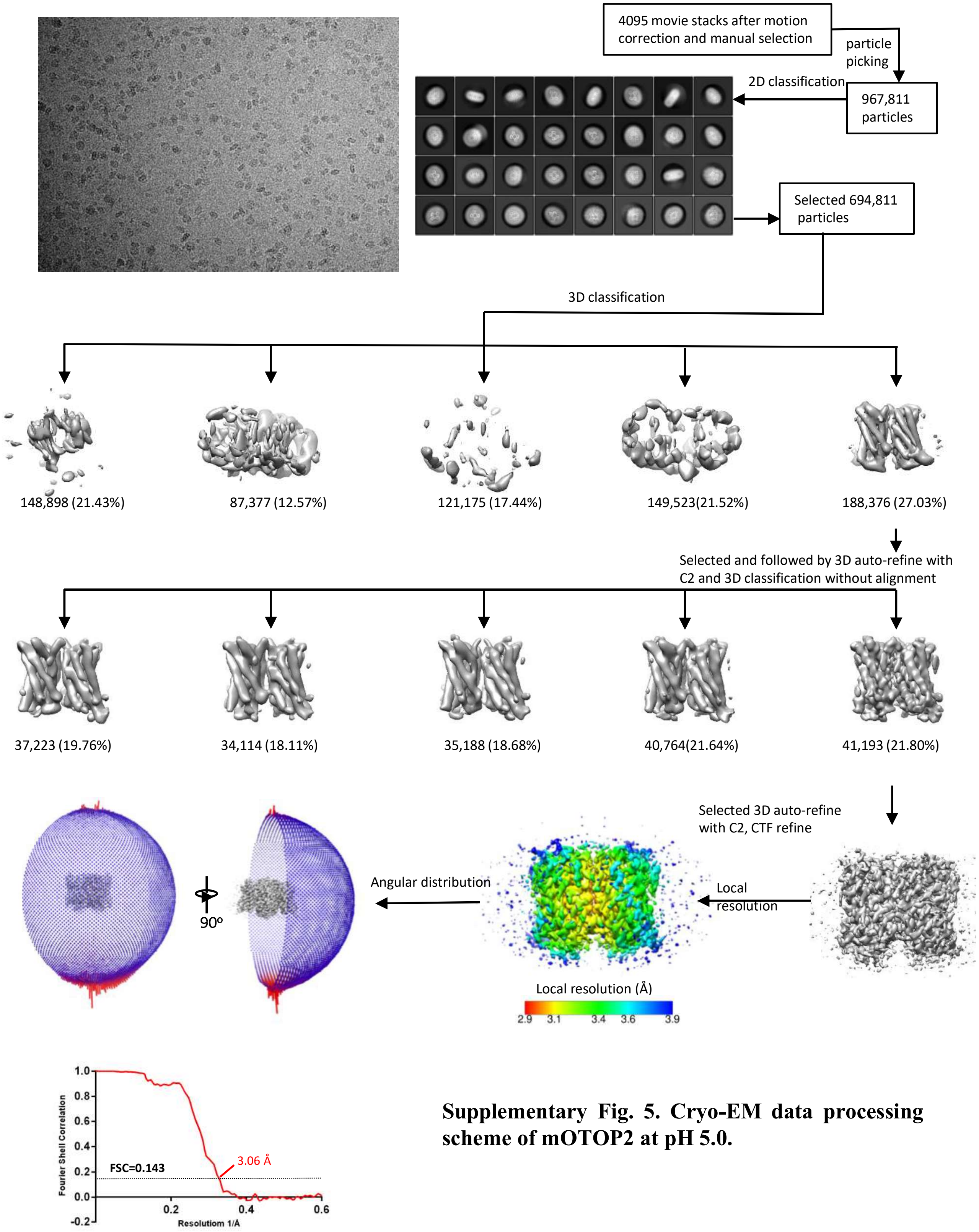

Supplementary Fig. 5. Cryo-EM data processing scheme of mOTOP2 at pH 5.0.

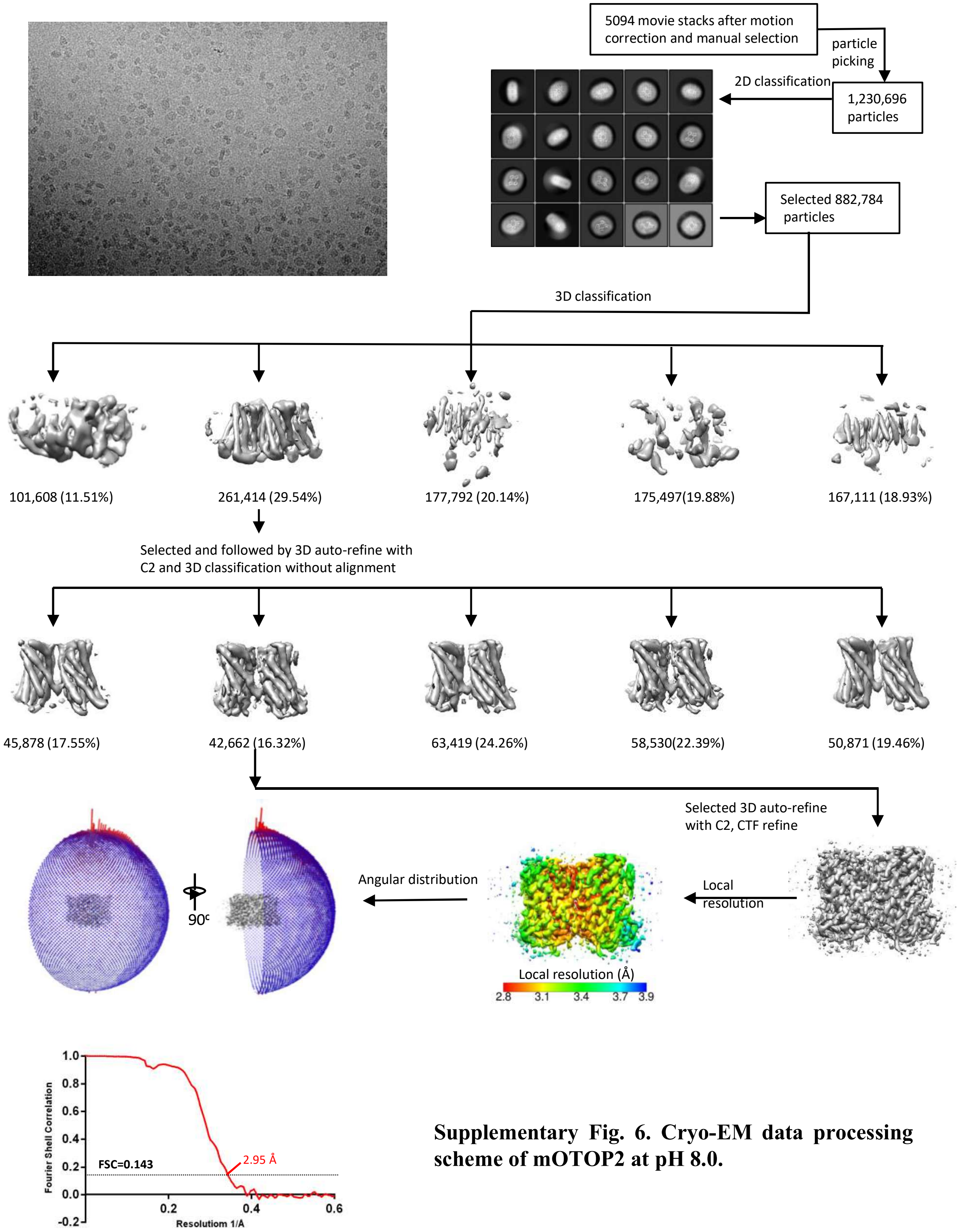

Supplementary Fig. 6. Cryo-EM data processing scheme of mOTOP2 at pH 8.0.

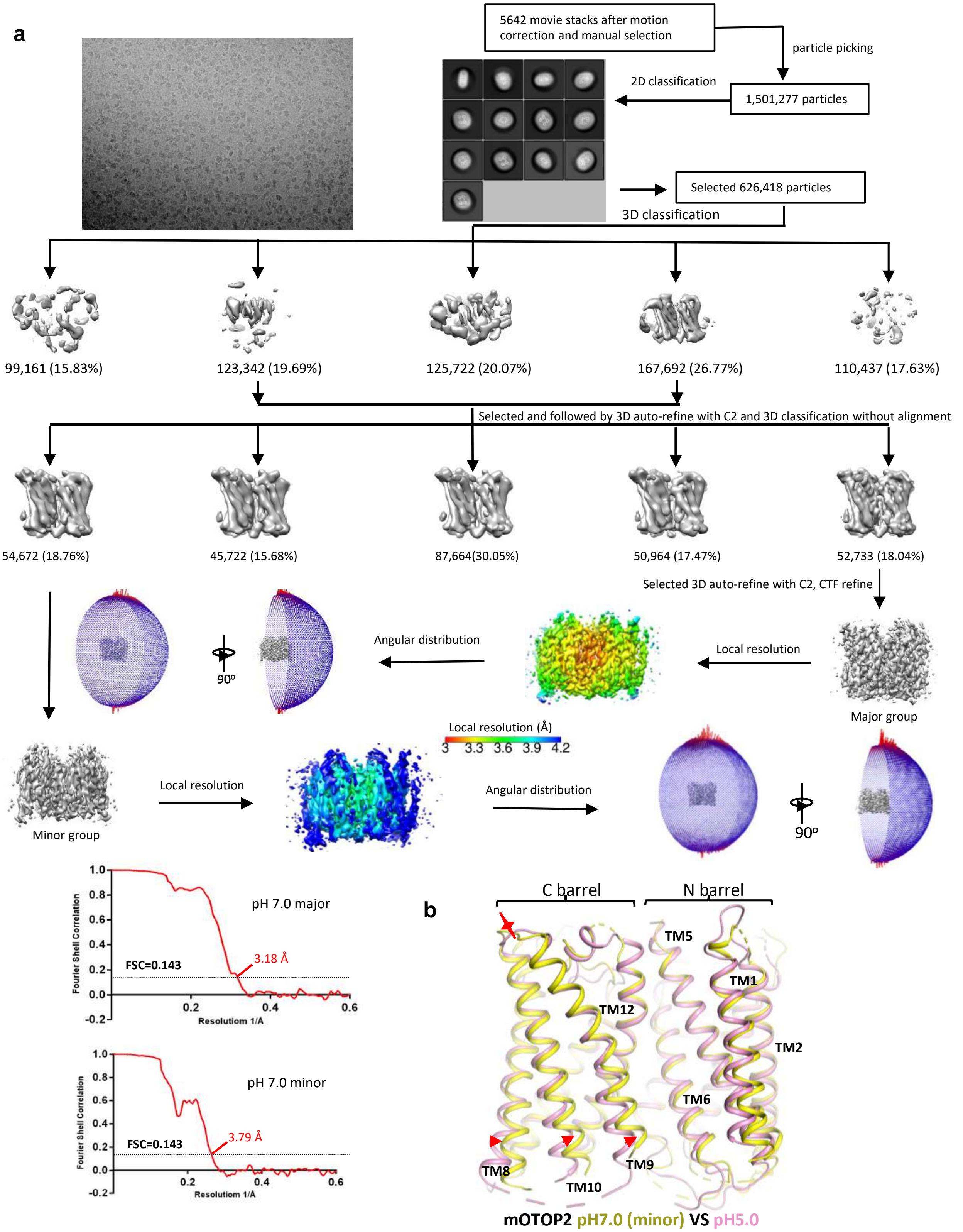

Supplementary Fig. 7. mOTOP2 intermediate state at pH 7.0

(**a**). Cryo-EM data processing scheme of mOTOP2 at pH 7.0.

(**b**). Structural comparison between mOTOP2 structures determined at pH 5.0 (pink) and at pH 7.0 from the minor group of particles (yellow).

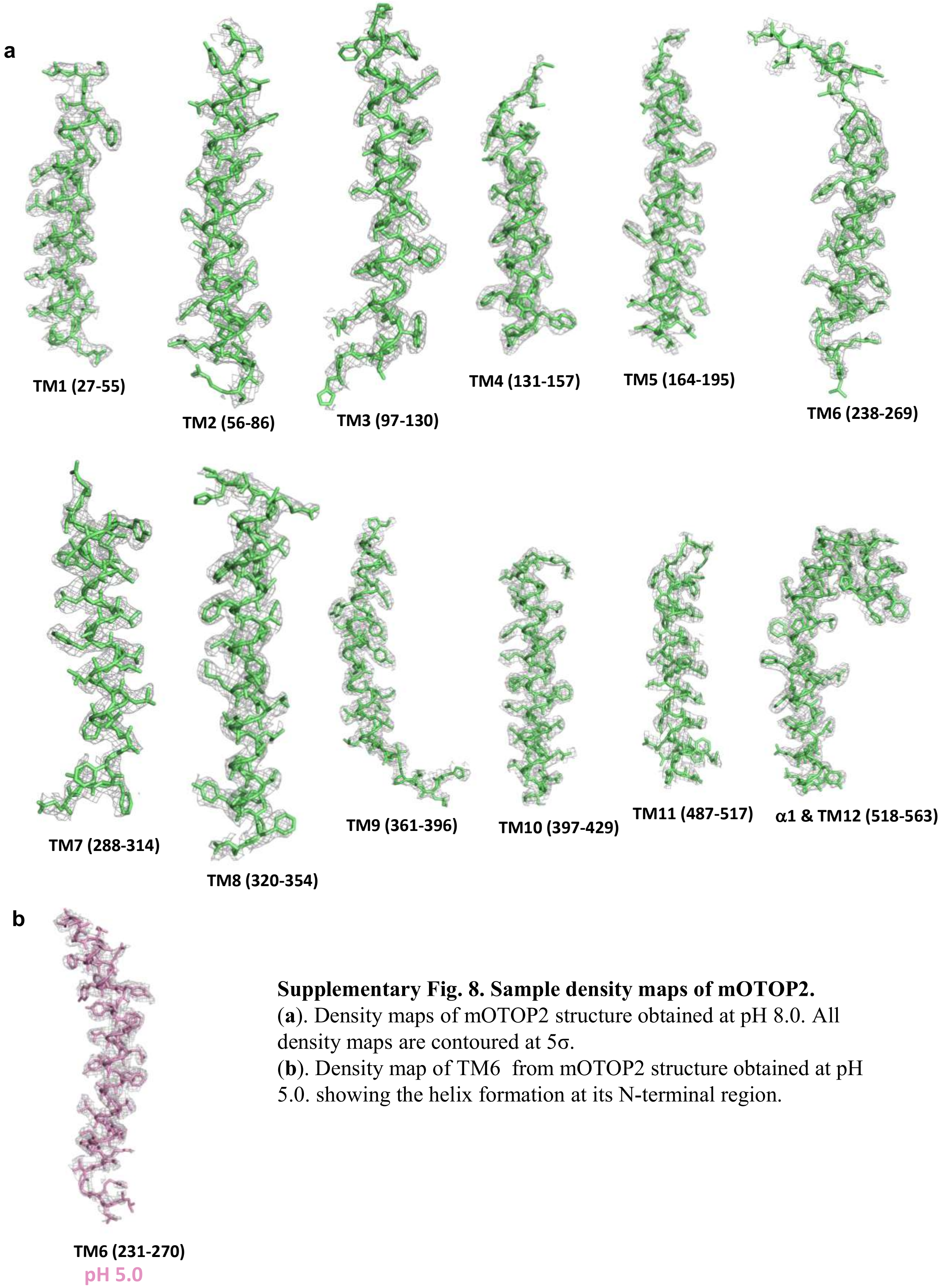

Supplementary Fig. 8. Sample density maps of mOTOP2.
(**a**). Density maps of mOTOP2 structure obtained at pH 8.0. All density maps are contoured at 5σ.
(**b**). Density map of TM6 from mOTOP2 structure obtained at pH 5.0. showing the helix formation at its N-terminal region.

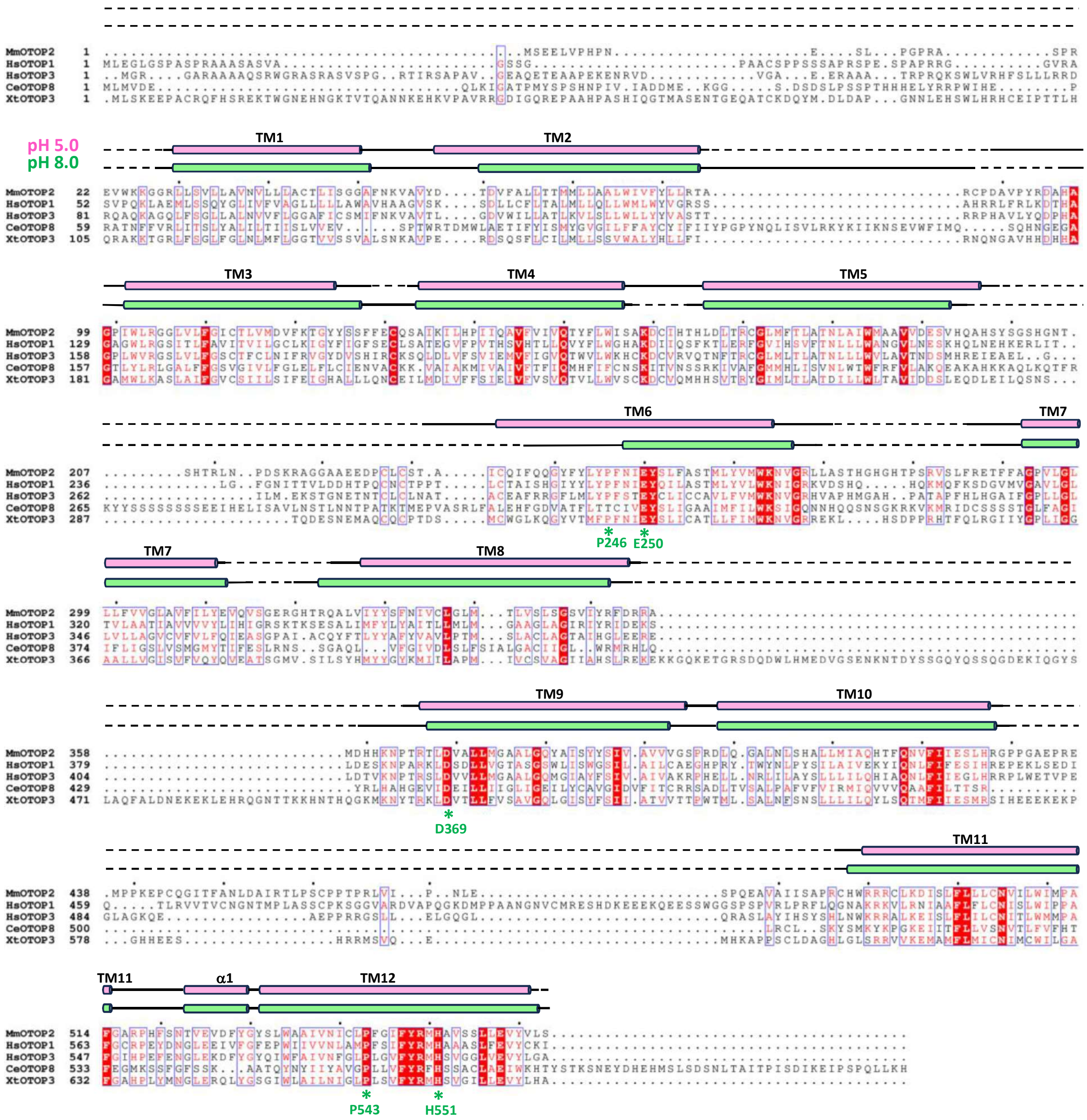

Supplementary Fig. 9. Sequence alignment and secondary structure assignments of mOTOP2. Conserved residues important for proton conduction are marked by green asterisks. The secondary structure assignments are colored pink and green for structures determined at pH 5.0 and 8.0, respectively.

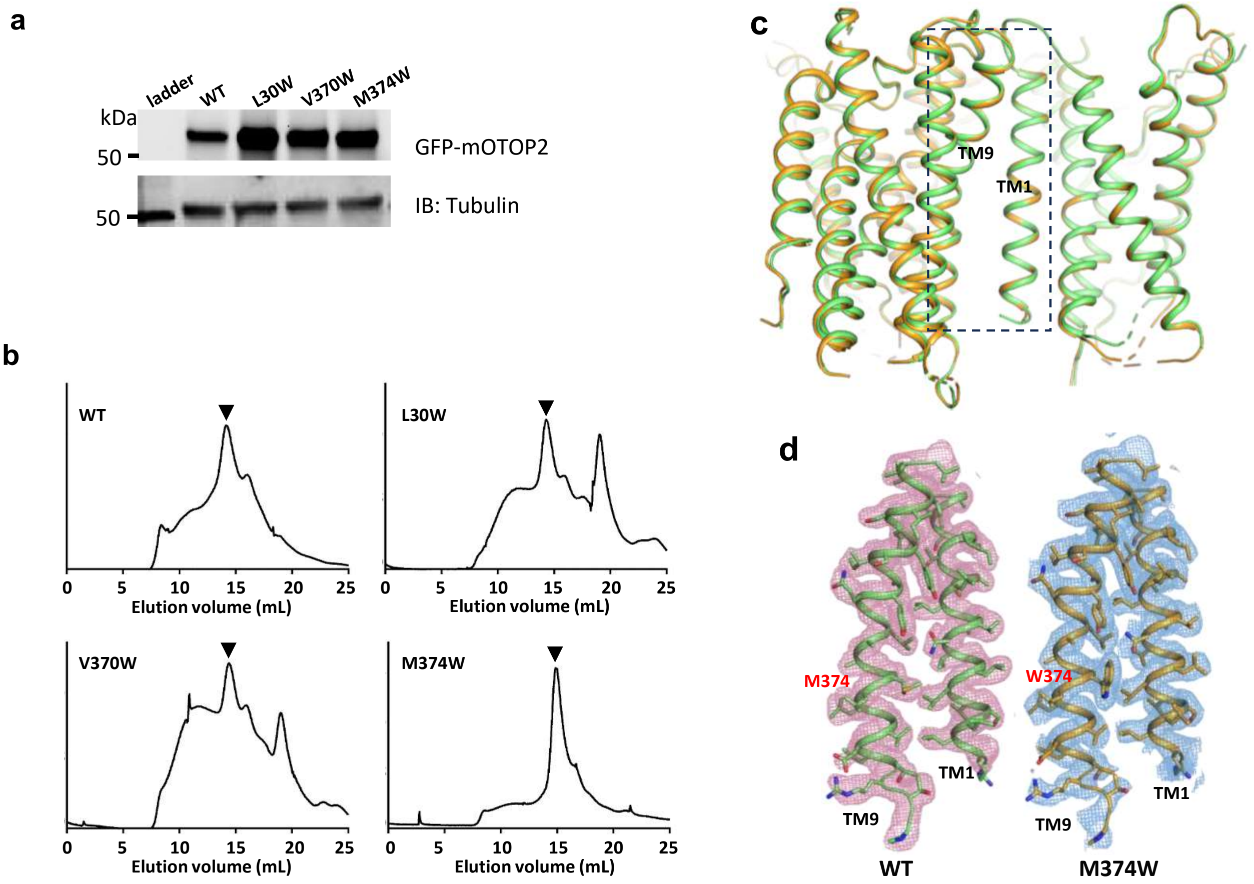

Supplementary Fig. 10. Expression, purification, and structures of mOTP2 and its loss-of-function mutants.

(**a**). Expression of GFP-tagged WT mOTOP2 and its loss-of-function mutants in HEK293 cells used for electrophysiological recordings.

(**b**). Gel filtration profiles of purified WT mOTOP2 and its loss-of-function mutants. Arrows mark the peaks of the channel dimers.

(**c**). Superimposition of WT mOTOP2 (green) and its M374W loss-of-function mutant (gold) structures determined at pH 8.0. Only the N-barrel from one subunit and the C-barrel from the other subunit are shown in the comparison.

(**d**). EM density maps (contoured at 5 σ) of TM1 and TM9 at the dimer interface (boxed in **c**) from the WT mOTOP2 structure (pink mesh) and its M374W mutant structure (blue mesh). The two structures are virtually identical except for the side chain of residue 374.

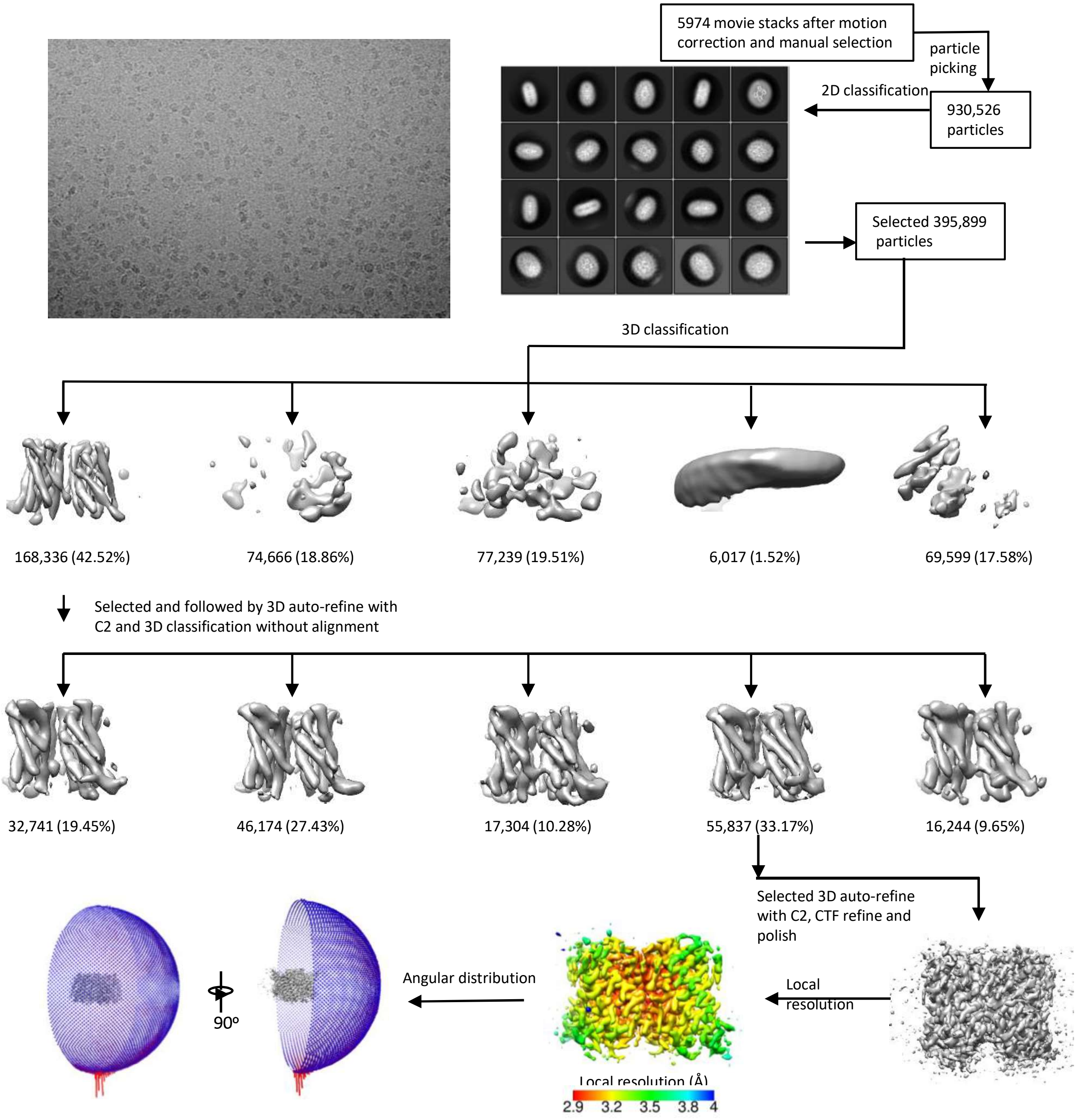

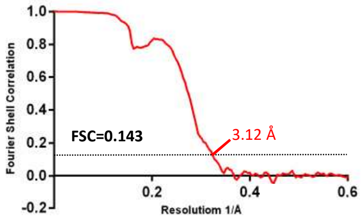

Supplementary Fig. 11 Cryo-EM data processing scheme of mOTOP2 M374W mutant at pH 8.0.

	CeOTOP8<br>EMDB-42214<br>PDB- 8UG5	CeOTOP8<br>EMDB- 42213<br>PDB- 8UG4	mOTOP2<br>EMDB- 42215<br>PDB- 8UG6	mOTOP2<br>EMDB-42216<br>PDB-8UG7	mOTOP2 (minor)<br>EMDB-42217<br>PDB-8UG8	mOTOP2 M374W<br>EMDB-42219<br>PDB-8UGA
**pH condition**	5.0	8.0	5.0	8.0	7.0	8.0
**Data collection and processing**						
Magnification	105,000	105,000	105,000	105,000	105,000	165,000
Voltage (kV)	300	300	300	300	300	300
Electron exposure (e–/Å2)	60	60	60	60	60	60
Defocus range (μm)	-0.9 - -2.2	-0.9 - -2.2	-0.9 - -2.2	-0.9 - -2.2	-0.9 - -2.2	-0.9 - -2.2
Pixel size (Å)	0.83	0.83	0.83	0.83	0.83	0.737
Symmetry imposed	C2	C2	C2	C2	C2	C2
Initial particle images (no.)	1,261,211	1,157,107	967,811	1,230,696	1,501,277	930,526
Final particle images (no.)	41,311	48,261	41,193	42,662	54,672	55,837
Map resolution (Å)	2.91	3.02	3.06	2.95	3.79	3.12
FSC threshold	0.143	0.143	0.143	0.143	0.143	0.143
**Refinement**						
Initial model used (PDB code)	6O84	6O84	6O84	6O84	6O84	6O84
Model resolution (Å)	2.91	3.02	3.06	2.95	3.79	3.12
FSC threshold						
Map sharpening B factor (Å2)	-73.19	-70.37	-99.18	-81.05	-159.103	-96.48
Model composition						
Non-hydrogen atoms	6,156	7,778	6,336	6,242	5,916	6,302
Protein residues	764	972	800	786	750	790
Ligands	0	0	0	0	0	0
B factors (Å2)						
Protein	49.23	56.34	55.66	59.93	96.35	60.56
R.m.s. deviations						
Bond lengths (Å)	0.008	0.006	0.004	0.005	0.003	0.004
Bond angles (°)	1.231	0.707	0.570	0.664	0.527	0.595
Validation						
MolProbity score	1.78	1.58	1.71	1.42	1.51	1.65
Clashscore	8.40	8.93	10.85	7.06	9.77	7.31
Poor rotamers (%)	0	0	0	0	0	0
Ramachandran plot						
Favored (%)	95.38	97.49	97.14	97.88	98.03	96.31
Allowed (%)	4.62	2.51	2.86	2.12	1.97	3.69
Disallowed (%)	0	0	0	0	0	0

Supplementary Table 1. Data collection and refinement statistics.
