# Supplementary material for: Structural mechanism of proton conduction in otopetrin proton channel
Source: Nat Commun. 2024 Aug 23;15:7250. doi: 10.1038/s41467-024-51803-x (PMC11343839; doi:10.1038/s41467-024-51803-x)
Supplement: Supplementary file 3 — Description of Additional Supplementary Files [file 41467_2024_51803_MOESM3_ESM.pdf]

Description of Additional Supplementary Files

File Name: Supplementary Movie 1

Description: Helix-to-loop transition at TM12 of CeOTOP8.
