# Supplementary figures and images for: Structural mechanism of proton conduction in otopetrin proton channel
Source: Nat Commun. 2024 Aug 23;15:7250. doi: 10.1038/s41467-024-51803-x (PMC11343839; doi:10.1038/s41467-024-51803-x)

Supplementary. Fig. 10 a

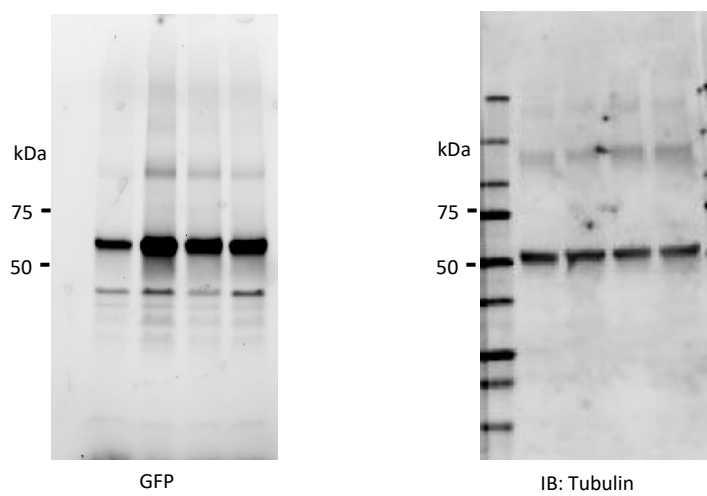

Supplement: Supplementary file 6 — Source Data [file 41467_2024_51803_MOESM6_ESM.pdf]
